# Q Fever: Who Is at Risk? A Serological Survey in the General Population and Occupationally Exposed Individuals in Northern Italy

**DOI:** 10.3390/pathogens14090869

**Published:** 2025-09-01

**Authors:** Alice Fincato, Laura Lucchese, Laura Bellinati, Elisa Mazzotta, Silvia Ragolia, Shirin Asa’Ad, Cristiano Salata, Alda Natale

**Affiliations:** 1 Istituto Zooprofilattico Sperimentale delle Venezie, SCT3 Padova, Vicenza e Rovigo, 35020 Legnaro, Italy; afincato@izsvenezie.it (A.F.); lbellinati@izsvenezie.it (L.B.); anatale@izsvenezie.it (A.N.); 2 Department Microbiology, Ospedale Ca’ Foncello Treviso, 31100 Treviso, Italy; silvia.ragolia@aulss2.veneto.it; 3 Department of Laboratory Medicine, Ospedale dell’Angelo Mestre, 30174 Venezia, Italy; shirin.asaad@aulss3.veneto.it; 4 Department of Molecular Medicine, University of Padua, 35121 Padua, Italy; cristiano.salata@unipd.it

**Keywords:** *Coxiella burnetii*, Q fever, serology, risk of exposure, one health

## Abstract

Background: Q fever is a zoonotic disease caused by the intracellular bacterium *Coxiella* (*C.*) *burnetii*. In ruminants, it mainly leads to reproductive disorders. In humans, transmission typically occurs through direct contact with infected animals or inhalation of contaminated aerosols. Although it is a notifiable disease in the European Union for both humans and certain animal species, the actual incidence is likely underestimated due to the non-specific nature of clinical symptoms. Domestic ruminants are considered the main reservoirs of *C. burnetii*, placing farmers and veterinarians at increased occupational risk of infection. Objectives: This study aimed to assess the risk of Q fever infection in northern Italy by comparing the seroprevalence rates between professionally exposed individuals and not professionally exposed people. Methods: A total of 209 serum samples were analysed: 117 from exposed professionals (veterinarians, biologists, agronomists, laboratory technicians) and 92 from professionally unexposed people (control group). Serum samples were tested with a commercial enzyme-linked immunosorbent assay to detect the presence of IgG against *C. burnetii*. Positive and doubtful samples were further investigated with a commercial immunofluorescence assay for detection of IgM and IgG. Epidemiological data were also collected to explore potential risk factors. Results: In total, 10 of the 117 exposed individuals tested positive, yielding a seroprevalence of 8.6%, while only 1 of the 92 control subjects tested positive (1.1%). These findings indicate a significantly higher occupational risk of *C. burnetii* infection among exposed professionals compared to the general population. Conclusions: The results highlight the need for preventive measures and surveillance in at-risk occupational groups.

## 1. Introduction

Q fever is a zoonotic disease caused by the obligate intracellular bacterium *Coxiella* (*C.*) *burnetii*, a small Gram-negative pathogen belonging to the class of Gammaproteobacteria, family Coxiellaceae [1,2]. *C. burnetii* is extremely resistant in the environment, can infect a wide range of animals, and the reservoirs include domestic farm animals such as goats, sheep, and cattle but also wild animals such as rodents, small mammals, wild ruminants, and non-mammalian species such as birds and reptiles [3,4]. It is still controversial whether the identification of the pathogen in ticks is related to their role as an environmental reservoir or as a vector [1,5,6,7]. Domestic ruminants represent the primary reservoir responsible for human *C. burnetii* infection. Infected ruminants, often asymptomatic, can shed large amount of bacteria through birth products, milk, urine, and faeces [8,9,10]. Consequently, the management of infected farms poses a significant occupational risk of infection for workers.

In humans, Q fever is associated with a wide spectrum of clinical manifestations ranging from asymptomatic cases to mild and severe illness (mainly pneumonia) and, in rare instances, severe chronic disease with potential fatal outcome [11,12,13]. Human infection with *C. burnetii* occurs mainly via inhalation of contaminated dust or contact with infected animal fluids, while consumption of contaminated raw dairy products, particularly from sheep, represents an additional potential transmission route [14,15,16,17,18,19,20,21,22,23,24]. Percutaneous exposure [25], transfusion [26], and sexual intercourse [27,28] are negligible routes of transmission. Human *C. burnetii* infection, formerly described as acute or chronic Q fever, is now classified as primary or persistent infection. Anti-phase 2 antibodies typically predominate during primary infection, while anti-phase 1 antibodies are associated with persistent infection, although serological responses can vary depending on the strain [29,30,31,32,33,34,35,36,37,38,39].

Several diagnostic methods are available for detecting *C. burnetii*: Polymerase chain reaction (PCR) has become the preferred method for direct diagnosis of *C. burnetii*, providing a rapid, safe, and more sensitive and specific alternative to conventional staining, using whole blood in the acute phase or biopsy specimens in persistent infection [40,41,42]. Serology is commonly employed to assess *C. burnetii* infection and population exposure: the immunofluorescence assay (IFA) is considered the reference method, while enzyme-linked immunosorbent assay (ELISA) and chemiluminescent immunoassay (CLIA) provide efficient options for large-scale screening; for seroprevalence studies, it is generally recommended to combine two independent serological methods. The complement fixation test (CFT) is less sensitive and more complex to perform. For seroprevalence studies, it is generally recommended to combine two independent serological methods, with seroconversion detectable 7–15 days after symptom onset and infection confirmed by a fourfold rise in phase 2 IgM or IgG titres between paired samples collected 3–6 weeks apart [43,44].

Despite the infection being endemic and well documented in ruminants, with a herd prevalence almost reaching 50% in dairy cattle farms [14,43,45,46,47,48], limited information is available on the serological prevalence of human Q fever in Italy. Indeed, existing data primarily pertain to the southern regions of the country, with the exception of four outbreaks in Northern Italy [48,49,50,51,52,53,54,55]. According to a most recent seroepidemiological study conducted in Sardinia on 1792 patients (4310 serum samples), an overall *C. burnetii* seroprevalence of 27% was reported, particularly associated with sheep and goat farming areas, confirming the endemic circulation of the pathogen and the risk of human exposure [53]. Furthermore, the latest human outbreaks in European countries such as Slovakia, the Netherlands, Germany, and Cyprus [56,57,58,59], as well as a recent outbreak among tourists in Northern Italy (Bozen) [52,60], highlight the need for reliable data on the distribution of *C. burnetii.*

The attention on Q fever in Europe increased dramatically after an outbreak occurred in the Netherlands between 2007 and 2010, with more than 4000 reported cases [58,61]. The spread and severity of human *C. burnetii* infection were linked to highly infected goat farms near urban areas, influenced by farm density, climatic conditions, and strain virulence [62,63,64].

The infectious dose of *C. burnetii* required to cause infection in humans via inhalation is extremely low. Individuals who come into contact with infected animals or animal products—such as farmers, veterinarians, veterinary assistants, and public health professionals—are considered at high risk of infection [65,66,67,68]. In addition, people who consume raw milk and dairy products are at moderate risk [20,23,65,69,70].

The aim of the present study was to compare the seroprevalence in a sample of occupationally exposed and unexposed Italian people, mainly from the north-eastern regions, to assess the risk of infection.

## 2. Materials and Methods

### 2.1. Sample Population

This cross-sectional study was conducted in the province of Padua, Italy. All participants provided written informed consent for inclusion in the study and agreed to the use of their personal data in accordance with the privacy policy, and the Italian Scientific Ethics Committee approved all procedures (n° 12—4589AO/18 (CESC)—AOP1560 (URC)—Prot. n. 0058197—02/10/2019). Licensed healthcare professionals collected human blood samples, and all serum samples were kept refrigerated during transport to the laboratory.

Participants were divided into two groups:

1. Professionally exposed group (EG): Individuals with occupational activities involving direct or indirect contact with *C. burnetii* or potentially contaminated materials. It included veterinarians (including veterinary students), agronomists, and laboratory technicians working with potentially *C. burnetii* contaminated samples. Samples for this group were collected in the context of two educational events organised by the Istituto Zooprofilattico Sperimentale delle Venezie, Legnaro, Padua (Italy), scheduled during a research project funded by the Italian Ministry of Health (RC IZSVE 16/2016).
2. Not professionally exposed group (NEG) or control group: Healthy individuals without occupational risk of *C. burnetii* exposure. The group consisted of blood donors, selected as a representative sample of the healthy general population. Blood samples were obtained during routine donation sessions, avoiding additional invasive procedures.

All the participants completed an epidemiological questionnaire (see Supplementary Materials File S1) to assess their exposure to potential risk factors for *C. burnetii* infection. The questionnaire included information on demographic characteristics (age and sex), place of residence (urban or rural), occupation (veterinarian, agronomist, technician, or other), and the frequency of exposure to domestic, synanthropic, or wild animals (including livestock, pets, and local fauna). Additional data were gathered on contact with livestock-derived materials, such as birth products, aborted foetuses, and animal products. The final section of the questionnaire addressed any previous experience of relevant clinical symptoms, including atypical febrile illness, endocarditis, hepatitis, and/or prolonged fatigue.

Regarding laboratory technicians in the professionally exposed group, only individuals handling samples with known or potential *C. burnetii* contamination were classified under this category.

Based on questionnaire responses, two individuals in the NEG were reclassified as professionally exposed due to their occupational activities: one was a farmer and the other assisted with family agricultural work.

### 2.2. Serological Analysis

A two-step protocol was employed for the serological analysis. First, all samples were screened for the presence of antibodies to *C. burnetii* using a commercial ELISA test. Samples that were positive/doubtful were then further tested using two commercial indirect immunofluorescence (IFI) assays. In addition, the immunofluorescence assays were also performed on samples that were negative in the ELISA tests but with optical density (OD) values close to those of doubt.

#### 2.2.1. ELISA Analysis

Commercial *C. burnetii* ELISA assays (Serion ELISA classic, Virion-Serion, Wüzburg, Germany) were used for the detection of Immunoglobulin G against phase 1 and phase 2 anti-*C. burnetii*. According to the manufacturer protocol, the samples were diluted 1:100 in sample diluent for phase 1 analysis and 1:500 for phase 2 analysis. All samples and controls/standards added to the microtiter plates were incubated for 60 min at 37 °C in a humidity chamber. After washing, conjugate was added to each well, except for the blank wells, and the plates were incubated for 30 min at 37 °C in a humid chamber. Finally, washes were performed as before. After 30 min at 37 °C with the substrate solution in the dark, the stop solution was added to each well. The OD at 405 nm was read within 15 min. Each analytical session included controls and standard to validate the sample results using the qualitative manufacturer protocol.

#### 2.2.2. IFI Analysis

The selected samples were tested using commercial indirect immunofluorescence (IFI) tests (Focus Diagnostic—Alifax, Legnaro, Italy) for the detection of IgM and IgG anti-*C. burnetii* in phases 1 and 2: the test panel included four reactions (IgG—phase 1, IgG—phase 2, IgM—phase 1, IgM—phase 2). Briefly, all samples were diluted 1:16 in the appropriate dilution buffer provided in the kit. The controls were ready to use, but the positive reference required a 1:8 dilution to assess the reliable cut-off. Samples and controls were applied to the dual-spot wells of the slides and incubated at 37 °C in a humidity chamber for 90 min for IgM detection and 30 min for IgG detection. Three washes in phosphate buffered saline solution and water were then required according to the manufacturer’s instructions. After the slides had dried, the conjugate was incubated for 30 min at 37 °C in a humidity chamber. Finally, three washes were performed. The cover-slipped slides were observed under a fluorescence microscope with immersion oil at 40× or 50×. The commercial controls were included in each analytical session. Samples positive at the screening dilution were tested after twofold dilution in phosphate buffered saline to determine the cut-off of positivity.

### 2.3. Statistical Analysis

Data were analysed using the open-source software R version 4.3.3 (https://www.R-project.org/ last access 1 July 2025). The seroprevalence of *C. burnetii* and the corresponding 95% confidence interval (CI) were calculated, and frequency distributions were calculated for the population characteristics and exposure variables. Pearson’s Χ^2^ test was used to compare the seroprevalence of *C. burnetii* between the professionally exposed and the unexposed groups, as well as across different exposure categories. In order to assess any correlation between *C. burnetii* seropositivity and the age of the participant, each person was categorised into an age group as follows: (i) people less than 25 years of age to represent a possible group of veterinarian students exposed to *C. burnetii*, (ii) people between 26 and 59 years of age to represent the adult group exposed in their occupation, (iii) people over 60 years of age to represent the older workers’ group, possibly more exposed over the years, but also due to habits less oriented toward biosafety. Generalized Additive Models (GAMs) were employed to assess the association between the predictor variables (e.g., age, occupation, exposure to animals) and the seropositivity. Odds ratios were calculated using the ‘mgcv’ package with binomial error (logit-link function). Results were considered significant at a *p* value < 0.05.

## 3. Results

Serum samples were collected from a total of 209 participants: 117 in the professionally exposed group and 92 in the not professionally exposed group. Of these, 11 participants tested seropositive for *C. burnetii*, corresponding to a seroprevalence of 5.3% (95% CI: 2.7–9.2). Among the positive samples, 10 were from the professionally exposed group, while only 1 positive sample was identified in the control group. Detailed serological results for each positive sample are presented in Table 1.

When stratified into groups, the seroprevalence was 8.6% (95% CI: 4.2–15.3) among professionally exposed individuals and 1.1% (95% CI: 0–5.8) among not professionally exposed. The difference in seroprevalence was statistically significant (*p* = 0.037).

The two groups were similar in sex distribution (*p* = 0.387) but differed in age (*p* = 0.018), with younger individuals predominating in the control group and a higher proportion of adults in the professionally exposed group. A higher proportion of professionally exposed individuals lived in rural areas (*p* < 0.001) and worked outdoors (*p* < 0.001). Conversely, participants in the not professionally exposed group more frequently reported consumption of raw animal products (*p* = 0.016). Additionally, the professionally exposed group reported a higher prevalence of current or past symptoms compatible with Q fever (*p* = 0.009). All questionnaire items related to occupational hazards indicated in the professionally exposed group a significantly more frequent contact with domestic, livestock, and wild animals (*p* < 0.001 for all), as well as their faeces, secretions, and associated environmental materials such as straw, hay, and dust.

Within the control group no demographic or behavioural factor was significantly associated with seropositivity, likely due to the low number of positives (*n* = 1). Among exposed individuals, seropositivity was more frequent in those who consumed raw animal products (*p* = 0.042) and in those with a history of tick exposure (*p* = 0.015).

A pooled Fisher’s exact test identified significant associations between seropositivity and occupational category, as well as variables directly related to occupational exposure, such as animal contact, handling of animal products, and environmental exposure (*p* < 0.001 for most). Sex and age lost significance when seropositivity was considered, suggesting that the age difference between groups was not related to infection status. However, residential area (*p* = 0.001), raw meat consumption (*p* = 0.005), and outdoor work (*p* < 0.001) remained significantly associated.

**Figure 1:**
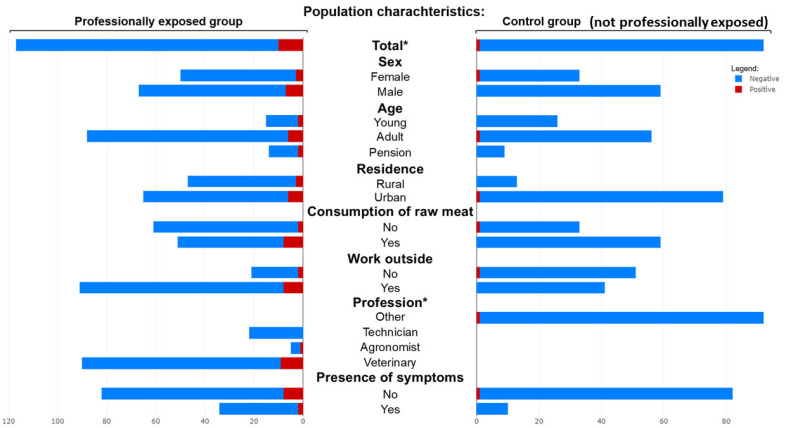
Butterfly plot showing the population characteristics of the exposed and control group, and results for each population. Stars indicate the significance level (* for *p* < 0.05) of the Pearson’s Χ^2^ test for each characteristic when comparing all seropositive and seronegative individuals across the entire study population.

**Figure 2:**
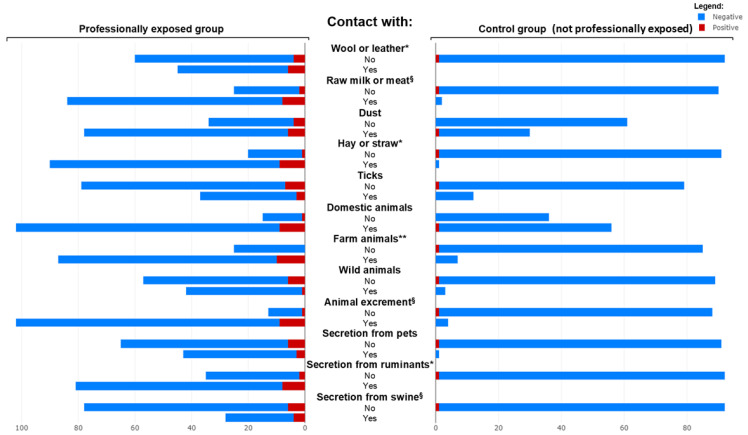
Butterfly plot showing the contact with potential risk factors of the exposed and control group, and results for each population. Stars indicate the significance level (** for *p* < 0.01, * for *p* < 0.05, § for *p* < 0.1) of the Pearson’s Χ^2^ test for each characteristic when comparing all seropositive and seronegative individuals across the entire study population.

When comparing all seropositive (*n* = 11) and seronegative (*n* = 198) individuals across the entire study population, seropositivity was significantly associated with being in the professionally exposed group rather than the control group (*p* = 0.025). Positive individuals reported more frequent contact with livestock (*p* = 0.003), worked more often in professions such as veterinary medicine (*p* = 0.032), and had higher exposure to animal-derived materials such as wool or hides (*p* = 0.019), straw or hay (*p* = 0.025), and ruminant secretions (*p* = 0.025). No significant differences were observed in sex, age, residence, and contact with domestic or wild animals, tick exposure, or most other occupational/environmental variables. Figure 1 summarizes the demographic and occupational differences between the professionally exposed and control groups, while Figure 2 illustrates the distribution of questionnaire responses regarding potential risk factors between the two groups.

GAMs indicated that individuals in the occupationally exposed group had approximately 8.5 times higher odds of being seropositive compared to those in the control group (*p* = 0.0431).

**Figure 3:**
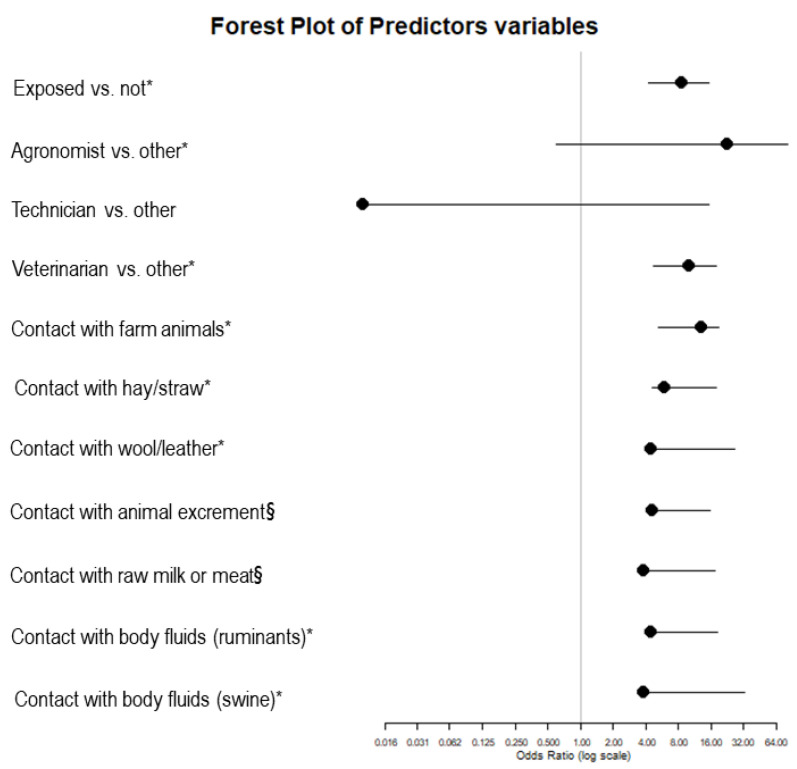
Forest plot showing odds ratios (OR) and 95% confidence intervals (CI) for demographic, occupational, and exposure-related predictors of *C. burnetii* seropositivity, based on GAMs. The vertical grey line indicates the null value (OR = 1). Circles represent point estimates (ORs), and horizontal lines represent the corresponding 95% CIs. Significant predictors included occupational exposure, profession (agronomist, veterinarian), and contact with farm animals, hay or straw, wool or leather, ruminant body fluids, and swine body fluids. Stars indicate the significance level (* for *p* < 0.05, § for *p* < 0.1).

Figure 3 shows the model results for the variables that were statistically significant in the analysis. Prevalence ratio, 95% confidence intervals and odds ratio of the risk analysis, and the *p* value are specified in Supplementary Materials File S2. The highest occupational risk of *C. burnetii* infection was observed among agronomists and veterinarians, with odds approximately 23-fold and 10-fold higher than the control group, respectively. In contrast, no laboratory technicians tested seropositive, and the difference in infection risk compared to the control group was not statistically significant.

When considering possible exposure to known risk factors across the entire study population, contact with farm animals was associated with the highest infection risk, corresponding to approximately 13-fold increased odds of seropositivity compared to individuals with no such contact. Additional significant associations were found for contact with hay or straw and with secretions from ruminants and pigs. Notably, contact with animal-derived products such as wool or leather was linked to a 4.5-fold higher risk of infection compared to those without such exposure.

When the model was fitted using multinomial analysis, none of the predictor variables remained statistically significant, indicating the presence of multicollinearity. Multicollinearity occurs when two or more predictor variables in a model are highly correlated, resulting in overlapping information and reduced model interpretability. Several of the identified risk factors, such as contact with animals, hay or straw, and secretions from ruminants and pigs, are likely to be interrelated. For example, individuals in frequent contact with farm animals are also more likely to be exposed to animal secretions and bedding materials, all of which are established risk factors for *C. burnetii* infection. The loss of statistical significance in the adjusted model underscores the challenge of isolating the individual effects of correlated exposure within this population.

## 4. Discussion

Q fever is a zoonotic disease in which wild and domestic animals are reservoirs of the pathogen and human cases can result from inhalation of contaminated aerosols or consumption of contaminated dairy products [31,36]. In humans, clinical manifestations range from no or mildly symptomatic cases to rare deaths [70,71].

In Europe, the implementation of Regulation (EU) 2016/429 (‘Animal Health Law’) mandates surveillance and notification for certain animal species, including *Bison* spp., *Bos* spp., *Bubalus* spp., *Ovis* spp., and *Capra* spp. Furthermore, Commission Implementing Regulation (EU) 2018/1882 lists Q fever among the diseases classified under category ‘E’. Moreover, in the EU, there are no harmonized rules or recommendations for the monitoring and reporting of Q fever in animals. Q fever is not explicitly listed in Annex I to Directive 2003/99/EC of the European Parliament and of the Council on the monitoring of zoonoses and zoonotic agents, amending Council Decision 90/424/EEC and repealing Council Directive 92/117/EEC [70]. Despite these legal provisions, the surveillance and reporting of *C. burnetii* infections in animals remain unharmonised across EU Member States. Similarly, notification of Q fever in humans is mandatory and must be reported to the Local Public Health Authority, with annual notifications submitted to the Regional Public Health Authority and the Ministry of Health. In Italy, recent papers report 17 human cases between 2015 and 2021 [6] and 14 human cases in a 2021 outbreak [52], while the annual report of the European Food Safety Agency (EFSA) and the latest EU report on zoonosis from the European Centre for Disease Prevention and Control (ECDC) report little or no data from Italy during same years [72]. This is likely attributable to differences among EU Member States, further complicating efforts to understand the complex epidemiology of Q fever. Consequently, the number of Q fever cases in both humans and animals is likely underreported and thus underestimated. Often, only outbreaks involving fewer than a few dozen people are reported, while isolated clinical cases are easily overlooked.

### 4.1. Study Objectives and Seroprevalence

This study aimed to evaluate the risk of Q fever infection by comparing the frequency of anti-*C. burnetii*-specific antibodies in occupationally exposed and non-exposed individuals in north-eastern Italy. To the best of the authors’ knowledge, no human serological data on Q fever have previously been reported for Northern Italy. Of the 209 samples collected, 11 were serologically positive for Q fever, resulting in an overall seroprevalence of 5.3% (95% CI: 2.7–9.2). Limited data are available from similar studies conducted in Europe, but reported rates tend to be higher [73,74]. Although the rates reported in the EFSA annual report appear lower [67], this is due to the inclusion of only those cases that resulted in official notifications from European member and non-member countries. Indeed, data on the prevalence of *C. burnetii* in people, animal, and products for human consumptions are limited and often influenced by variability in sampling, testing, and analytical methods used. Indeed, surveillance for Q fever is often implemented only following outbreaks in humans [13,75,76].

### 4.2. Exposure Assessment

All participant completed a questionnaire on lifestyle and exposure to known risk factors previously identified [77]. Occupational status was used to distinguish between the occupationally exposed and control groups. The seroprevalence rate in the professionally exposed group was 8.6% (95% CI: 4.2–15.3), compared to 1.1% in the non-exposed control group (95% CI: 0–5.8). Therefore, individuals in the occupational exposure group were 8.5 times more likely to be seropositive compared to those in the control group.

Within the occupationally exposed group, veterinarians and agronomists exhibited the highest risk of infection, with odds approximately 10 and 23 times higher, respectively, compared to the control group. In contrast, no laboratory technicians tested seropositive for *C. burnetii*, and no statistically significant difference was observed between this subgroup and the controls. These findings suggest that the standard biosafety measures implemented for laboratory personnel are effective in preventing accidental exposure to *C. burnetii*, even when handling potentially positive samples.

### 4.3. Risk Factor Analysis

Contact with farm animals represented the strongest risk factor (~13-fold higher odds). Other significant associations included exposure to hay or straw, ruminant and swine secretions, and animal products (wool, leather). Adjusted multinomial analysis showed no individual variable remained significant, indicating multicollinearity and the interrelated nature of risk factor [78,79].

### 4.4. Study Limitation and Future Perspectives

Limitations include small sample size and difficulty recruiting exposed individuals, as well as relative homogeneity of the occupational group, which may limit generalizability. Further studies and targeted screening programs, particularly among high-risk occupational groups, are warranted to better characterize the frequency of worker exposure and to identify the circulating *C. burnetii* strains. Such efforts would enhance our understanding of the epidemiological dynamics of Q fever and support more effective prevention and control strategies.

## 5. Conclusions

Q fever is a globally significant zoonotic disease that impacts both public and veterinary health and imposes substantial socioeconomic burdens on the livestock industry.

Public health strategies aimed at reducing the risk of infection, particularly among high-risk occupational groups, could contribute to lowering the incidence of human cases. Such measures have been clearly expressed by EFSA (https://storymaps.arcgis.com/stories/7f9d9bc1eeee4b838eaaa0d2576ee0c0 last access 1 July 2025) and include the use of protective equipment by farmers, farm technicians, and veterinarians. Proper manure management, awareness of transmission routes, preventive measures, and vaccination of animal reservoirs are key strategies to control *C. burnetii* infection and reduce human exposure. In conclusion, our findings underscore the significant role of occupational and environmental exposures in human infection. These results highlight the importance of continued epidemiological surveillance, targeted preventive measures for at-risk populations, and public health interventions to mitigate the burden of Q fever.

## Figures and Tables

**Table 1:** Results of serological analyses for individuals tested with IFI.

ID	ELISA IgG		IFI IgG		IFI IgM	
	IgG—Phase 1	IgG—Phase 2	IgG—Phase 1	IgG—Phase 2	IgM—Phase 1	IgM—Phase 2
EG 20	POS	POS	1:64	1:64	neg	neg
EG 26	POS	POS	1:256	1:256	neg	neg
EG 30	POS	POS	1:512	1:1024	1:256	1:256
EG 33	POS	POS	1:256	1:256	neg	neg
EG 90	POS	DOUB	1:128	1:128	neg	neg
EG 99	POS	POS	1:1024	1:512	neg	neg
EG 108	neg	DOUB	1:64	1:256	1:64	neg
EG 109	neg	neg	1:128	1:32	neg	neg
EG 115	neg	DOUB	1:128	1:64	neg	neg
EG 116	neg	POS	1:64	1:128	neg	neg
NEG 78	neg	DOUB	1:32	1:128	neg	neg

EG: professionally exposed group; NEG: not professionally exposed group; POS: positive, DOUB: doubtful result, neg: negative.

## Data Availability

Metadata are available from the authors following a reasonable request. Preliminary data have been presented as conference paper “Indagine sierologica su Febbre Q nell’uomo in categorie a rischio”, Lucchese L., Raoult D., Marangon S., Mion M., Giurisato I., Barberio A., Lonardi U., Natale A., XV Congresso Nazionale SIDILV, Monreale, 23–25th October 2013, pp. 314–315 [80].

## Supplementary Materials

The following supporting information can be downloaded at https://www.mdpi.com/article/10.3390/pathogens14090869/s1. File S1: Epidemiological questionnaire; File S2: Prevalence of the risk factor that were statistically significant to the fitted generalised linear mixed model.
