# Supplementary material for: Q Fever: Who Is at Risk? A Serological Survey in the General Population and Occupationally Exposed Individuals in Northern Italy
Source: Pathogens. 2025 Sep 1;14(9):869. doi: 10.3390/pathogens14090869 (PMC12472743; doi:10.3390/pathogens14090869)
Supplement: Supplementary file 1 [file pathogens-14-00869-s001.zip › Supplementary S1.Epidemiological questionnaire.pdf]

QUESTIONARIO

Codice Identificativo Univoco ______________________ Data________________

VALUTAZIONE DELLA DIFFUSIONE DELL'INFEZIONE CAUSATA DA COXIELLA BURNETII IN CATEGORIE PROFESSIONALI A RISCHIO E NELLA POPOLAZIONE UMANA NORMALE

Data di nascita: ________/_______ /________

Sesso: □ Maschio □ Femmina

Abita in ambiente : □ Urbano □ Rurale/silvestre

Al momento del prelievo gode di buona salute (assenza malattie acute)? □ Si □ No

Soffre di malattie neoplastiche o infiammatorie? □ Si □ No

Se si, specificare ____

Professione □Veterinario Libero professionista; □Veterinario dipendente SSN; □Agronomo; □altro…………..

Possiede animali domestici? □ no □ sì (indichi quali) ……………………………………………………

Possiede animali d'allevamento? □ no □ sì (indichi quali) ………………………………………….

Ha contatti con:_animali domestici □ no □ a volte □ spesso
Se sì, indicare le 3 specie principali……………………………………….
_animali d'allevamento □ no □ a volte □ spesso
Se sì, indicare le 3 specie principali……………………………………
_animali selvatici □ no □ a volte □ spesso
Se sì, indicare le 3 specie principali……………………………………

Svolge attività che prevedono il contatto con animali quali:
□ caccia
□ equitazione
□ falconeria
□ nessuna delle precedenti
□ allevamento volatili da cortile/ornamentali, indicare le specie……………
□ altro ……………………………………

E' mai stato morso da zecche? □no □sì
Se sì, indichi in quale regione……………………………………………………………………………..

Ha contatti con animali che presentano infestazioni da zecche? □ no □ a volte □ spesso
Se sì, indichi la specie animale ……………………………………………………………………………...

Consuma latte crudo, prodotti a base di latte crudo, carni crude? □no □a volte □spesso

Lavora in ambienti polverosi? □ no □ a volte □ spesso
Svolge lavori all'aperto? □ no □ a volte □ spesso

Nella sua attività lavorativa, entra in contatto con:
_paglia ☐ no ☐ a volte ☐ spesso
_fieno ☐ no ☐ a volte ☐ spesso
_lana grezza ☐ no ☐ a volte ☐ spesso
_pelli animali ☐ no ☐ a volte ☐ spesso
_deiezioni animali ☐ no ☐ a volte ☐ spesso
_latte crudo ☐ no ☐ a volte ☐ spesso
_carni crude ☐ no ☐ a volte ☐ spesso

E’ mai entrato in contatto con secrezioni vaginali, invogli fetali a seguito di parti/aborti?
_bovini ☐ no ☐ a volte ☐ spesso
_ovi-caprini ☐ no ☐ a volte ☐ spesso
_suini ☐ no ☐ a volte ☐ spesso
_cani ☐ no ☐ a volte ☐ spesso
_gatti ☐ no ☐ a volte ☐ spesso

E’ mai stato interessato da:
_Sindromi febbrili atipiche ☐ no ☐ sì
_Polmoniti atipiche ☐ no ☐ sì
_Epatiti atipiche ☐ no ☐ sì
_Endocardite ☐ no ☐ sì

Se sì, è stato sottoposto ad accertamento per *C. burnetii* o *Chlamydia spp.*? ☐ no ☐ sì

E’ affetto da problemi cardiaci/epatici/intestinali/ articolari cronici? ☐ no ☐ sì

Se si, specificare quali……………………………………………………

Soffre di sindrome da affaticamento cronico? ☐ no ☐ sì

Se donna, è mai stata interessata da aborto spontaneo? ☐ no ☐ sì (indicare se precoce o tardivo)……………………………………….

Le è mai stata diagnosticata positività per *C. burnetii* o *Chlamydia spp.*? ☐ no ☐ sì
Se sì, indicare brevemente in che occasione
……………………………………………………………………………………………………
……
……
……

Se desidera aggiungere informazioni che ritiene utili:
____
____
____
____

Grazie!
