# Supplementary material for: Q Fever: Who Is at Risk? A Serological Survey in the General Population and Occupationally Exposed Individuals in Northern Italy
Source: Pathogens. 2025 Sep 1;14(9):869. doi: 10.3390/pathogens14090869 (PMC12472743; doi:10.3390/pathogens14090869)
Supplement: Supplementary file 1 [file pathogens-14-00869-s001.zip › Supplementary material 2.docx.pdf]

Supplementary Material 2.

Prevalence of the risk factor that were statistically significant to the fitted generalised linear mixed model. Response rate indicate the number of participants that answer to the specific question. Prevalence ratio (PR) and 95% confidence intervals (CI95%) of seropositivity was calculated. For each risk factor the odds ratio, calculated as exp (Estimate) of the risk analysis, represent the odds of the outcome occurring given the predictor variable (Ref.). The p value is indicated.

Predictor	Response rate 100 % – n=209	PR - CI95%	Odds Ratio	p value
**Type**	100%			
Not exposed		1.1% CI95% 0 – 5.9	Ref.	
Exposed		8.5% CI95% 4.2 – 15.2	8.51	0.0431*
**Profession**	100%			
Other		1.1% CI95% 0 – 5.9	Ref.	
Agronomist		2.5% CI95% 0.6 – 80.6	22.79	0.0377*
Technician		0% CI95% 0 – 15.4	0.00	0.9887
Veterinary		10% CI95% 4.7 – 18.1	10.12	0.0298*
**Contact with**				
**Farm animals**	98%			
No		0.9% CI95% 0 – 5.0	Ref.	
Yes		10.6% CI95% 5.2 – 18.7	12.99	0.0155*
**Hay or straw**	96.5%			
No		1.8% CI95%0.2 – 6.4	Ref.	
Yes		9.9% CI95% 4.6 – 17.9	5.98	0.0245*
**Wool or leather**	94%			
No		3.3% CI95% 1.1 – 7.5	Ref.	
Yes		13.3% CI95% 5.1 – 26.8	4.52	0.0169*
**Animal excrement**	99%			
No		2.0% CI95% 0.2 – 7.0	Ref.	
Yes		8.5% CI95% 4.0 – 15.5	4.59	0.0551§
**Raw milk or meat**	96%			
No		2.6% CI95% 0.5 – 7.4	Ref.	
Yes		9.3% CI95% 4.1 – 17.5	3.83	0.0527§
**Body fluids (ruminants)**	99.5%			
No		2.4% CI95% 0.5 – 6.7	Ref.	
Yes		9.9% CI95% 4.4 – 18.5	4.53	0.0292*
**Body fluids (swine)**	95%			
No		4.1% CI95% 1.7 – 8.3	Ref.	
Yes		14.3% CI95% 4.0 – 32.7	3.88	0.0411*

Stars indicate the significance level (* for $p < 0.05$, § for $p < 0.1$).
